# Trends in the number and the quality of trial protocols involving children submitted to a French Institutional Review Board

**DOI:** 10.1186/s12874-017-0395-4

**Published:** 2017-08-23

**Authors:** Isabelle Gautier, Perrine Janiaud, Nelly Rollet, Nicolas André, Michel Tsimaratos, Catherine Cornu, Salma Malik, Stéphanie Gentile, Behrouz Kassaï

**Affiliations:** 1 Centre d’investigation Clinique Pédiatrique, INSERM CIC 9502, Hôpital d’Enfants de la Timone, AP-HM, 264, rue Saint-Pierre, 13005 Marseille, France; 2 EA3279 - Santé Publique: Maladies Chroniques et Qualité de Vie, Aix-Marseille Université, 13385 Marseille, France; 3 Evolutive Biology and Biometric Laboratory UMR5558 CNRS, Université Claude Bernard Lyon 1, 8 rue Guillaume Paradin, BP8071, 69376-CEDEX-08 Lyon, France; 4 EPICIME, Centre d’Investigation Clinique, INSERM CIC 1407, Hospices Civils de Lyon, 28 Avenue du Doyen Lépine, 69677-CEDEX Bron, France

**Keywords:** European Pediatric Regulation, Institutional Review board, Randomized Clinical Trials

## Abstract

**Background:**

There is a great need for high quality clinical research for children. The European Pediatric Regulation aimed to improve the quality of clinical trials in order to increase the availability of treatments for children. The main purpose of this study was to assess the evolution of both the number and the quality of pediatric trial protocols that were submitted to a French Institutional Review Board (IRB00009118) before and after the initiation of the EU Pediatric Regulation.

**Methods:**

All protocols submitted to the IRB00009118 between 2003 and 2014 and conducting research on subjects under eighteen years of age were eligible. The quality of randomized clinical trials was assessed according to the guidelines developed by the *Enhancing the QUAlity and Transparency Of health Research* (EQUATOR) Network and ranked using the Jadad score.

**Results:**

Out of 622 protocols submitted to the Institutional Review Board (IRB), 21% (133/622) included children. Among these 133 pediatric protocols, the number of submitted pediatric protocols doubled between the two studied periods. From 2003 to 2008, 47 protocols including 21 institutionally sponsored were submitted to the IRB and from 2009 until 2014, 86 protocols including 48 institutionally sponsored were submitted. No significant trend was observed on the quality of RCTs. The overall median score of RCTs on the Jadad scale was high (3.5), 70.0% of protocols had a Jadad score ≥ 3, and 30.0% had a score < 3.

**Conclusion:**

Following the EU Pediatric Regulation, the number of pediatric protocols submitted to the IRB00009118 tends to increase, but no change was noticed regarding their quality.

## Background

Using off-label or unlicensed drug in children is common worldwide. It has been reported that more than 50% of the interventions used in children do not rely on data from randomized controlled trials unlike adults [1]. Consequently, up to 80% of the prescriptions for children are either off-label or unlicensed [2]. Despite the great need for pediatric clinical research, randomized controlled trials (RCTs) which enroll children represent only 14% of all RCTs [3]. Over the last twenty years, the number of published RCTs regarding the pediatric population, compared with adults, did not increase [4, 5].

In addition, the low quality RCTs in children is a major concern as the treatment benefit may be overestimated. For instance, Moher et al. have highlighted that poor quality studies exaggerated the treatment benefit by 34% compared to high quality studies (ROR 0.66, 95% CI [0.52–0.83]) [6]. Weaknesses in the design, conduct and analysis of clinical trials, may therefore lead to misleading results [7–9].

Using online data banks to see the trends in terms of publication and number of pediatric clinical studies listed in the world on a given period may bring some insightful information. Thus, the quality of RCTs has been assessed in various fields of pediatrics [10–22]. In 2002, a systematic review evaluating the quality of published RCTs in pediatrics in complementary and alternative medicine [16], found that 81.3% (204/251) reported unclear allocation concealment, with no significant change over time. Inadequate method of random allocation, inappropriate description of outcome measures, inclusion or exclusion criteria, and small sample sizes are the main pitfalls that have been reported [17, 23–25]. Elsewhere, a review of the literature that analyzed the pediatric RCTs between 1948 and 2006 showed a global improvement in the quality of the methodology in 37.7% of all RCTs, but no description of the blinding and concealment methods were provided [19]. To overcome this issue, the EU has set a new Pediatric Regulation (No. 1901/2006) [26] that came into force in January 2007. It aims at “*achieving high quality ethical pediatric clinical research, to increase availability of authorized medicines that are appropriate for children and to produce better information on medicines*”. It resulted in the obligation for pharmaceutical companies to submit a Paediatric Investigation Plan for all drug development [1]. In addition, all pediatric labeling is rewarded with a 6-months patent extension. The expectations were to have safety and efficacy data for all pediatric age groups, robust data supporting new pediatric labeling and appropriate pediatric formulations. Although an increase in the quantity of RCTs has been observed, there are still large discrepancies between the trials conducted and the pediatric therapeutic needs [27].

The authorization to conduct a biomedical research (whether for a drug or not), intended to be used for application for market authorization, requires that both an Institutional Review Board (IRB) and drug regulation agency give their approval. In France, sponsors are required to submit their protocols to one of the 40 existing French IRBs. The protocol is usually submitted to the IRBs of the region where the principal investigator is registered. The main role of the board is to assess whether all scientific, ethical and legal requirements for conducting a research with human subjects are met.

The primary objective of our study was to assess the trends in the number and the quality of pediatric clinical trial protocols submitted to the French IRB00009118 before and after the implementation of the European Pediatric Regulation. The IRB00009118 reviews research protocols of the second biggest pediatric hospital in France.

## Methods

### Search strategy

We identified all protocols submitted to the IRB00009118 from January 2003 to December 2014. The Information submitted to the IRB is in paper format and contains the protocols, the investigator brochure, the investigators identities and training, insurance for patients and the authorization to conduct the research as a research facility if it is taking place in an unspecialized health center.

### Inclusion criteria

We included protocols that aim to conduct research on children aged 18 years or younger. Protocols recruiting both adults and children were also included. Only the first submitted versions of ultimately approved protocols were eligible.

### Data extraction and coding

Three authors (IG, PJ, NR) read each protocol, extracted and coded all the data based on a case report form (CRF). Another author (BK) resolved coding problems detected by the authors. The following characteristics were collected for all included protocols: administrative data (year of submission), design (parallel groups or cross over), statistical method (sample size calculation, mention of necessary items for sample size calculation, interim analysis, subgroup analysis, intention to treat analysis), randomization (allocation schemes: simple, blocked, stratified, adaptive and method of allocation concealment: sequentially numbered, opaque, sealed envelopes, pharmacy controlled, central randomization), level of blind (double-blind, simple blind, open-label) [28], and population (gender).

The quality of the protocols was assessed and coded according to the available guidelines developed by the EQUATOR-Network (Enhancing the QUAlity and Transparency Of health Research) [29] and more specifically the SPIRIT 2013 statement (Standard Protocol Items: Recommendations for Interventional Trials) for randomized clinical trials [30]. Items of the checklists that were not reported in the protocol were considered as missing and violated. RCTs were then scored using the Jadad score [28]. The Jadad score provides a quantitative estimation of the quality of randomized trials and can be replicated easily. The Jadad score is a five-point-scale including three criteria: randomization, level of blinding and mention of drop-outs and withdrawals. One point is attributed for the presence of each criterion. Points are added if randomization and double blinding are described and appropriate (one point each), or deducted if inappropriate. Out of the five potential points, high quality protocols are scored ≥3 and low quality <3. When the studies were non-blinded, we collected the data on the possible blind assessment of the outcome.

### Search in data banks

To confirm the trends observed in the number of our included protocols, data banks (PubMed and ClinicalTrials.gov) were also searched. The total number each year of studies that included children (birth-17) and submitted between 01/01/2005 to 12/31/2014 was extracted from the ClinicalTrials.gov database. The advanced search tool was used. Then, the number of drug trials each year was extracted by selecting from our file: “drug” and “intervention”.

On PubMed, all trials involving children were searched using the following search query: (clinical trial [MeSH Terms]) AND child [MeSH Terms] filter: publication dates: from 2005/01/01 to 2005/12/31 and this for every year until 2014. Then, the number of publications of drug trials involving children were searched using the following search query: (drug therapy [MeSH Major Topic]) AND child [MeSH Terms] filter: Article types: clinical trial and publication dates: from 2005/01/01 to 2005/12/31 and this for every year until 2014.

### Statistical analysis

Main characteristics of the protocols were described and compared using the Pearson’s chi2 test for qualitative variables and the Student test for quantitative ones. For RCTs, the median and interquartile range of Jadad scores were calculated. In order to show the temporal trends, the protocols were grouped in 6-years intervals by setting the cutoff year to 2008. RCTs protocols were also categorized as drug and non-drug studies.

In order to analyze the trends of the Jadad score over the years, we used the Mann–Whitney method with the time periods as the independent variable. The level of significance was set at a *p*-value <0.05. We tested the overall significance between the two periods. All statistical analyses were performed using the SPSS® 20.0 software.

### Sensitivity analysis

We initially performed an analysis including all randomized studies. Then a sensitivity analysis of the Jadad score was carried out by excluding open-label trials for which the lack of blinding was justified. For example, when the routes or the timing of drug administrations were different between the two groups, when a drug intervention was compared with surgery, or when the taste of a drug was difficult to mask, we considered that blinding conditions were not feasible. Since the Jadad score depends on the level of blinding of the study, these open studies usually had a lower Jadad score than those carried out in blind conditions. This sensitivity analysis was performed in order to check the robustness of our results regarding the quality of RCTs.

## Results

Between 2003 and 2014, 622 protocols were submitted to the IRB00009118. Twenty-one percent (133/622) of them enrolled children. Among them, the number of protocols including adults was greater than that of protocols including only children (56 children versus 77 adults and children protocols). Based on the French law of 9 August 2004 on Public Health Policy, 73% (97/133) of pediatric studies were considered “interventional”, 4% (5/133) “observational”, and 23% (31/133) “research on routine clinical care” (Table 1). Forty-eight percent of all protocols (64/133) were sponsored by pharmaceutical companies and 52% (69/133) by academic institutions. The comparison of the characteristics of protocols submitted before and after the regulation is provided in Tables 1 and 2.

**Table 1:** Characteristics of included protocols

Characteristics of all protocols	*n* = 133	2003–2008 (6 y) *n* = 47		2009–2014 (6 y) *n* = 86		*p*-value
	Total	Drug	Non drug	Drug	Non drug	
	n (%)	27 (57.5)	20 (42.5)	43 (50)	43 (50)	
Type of study						
Biomedical research	97 (72.9)	26	12	40	19	0.231
Routine health care	31 (23.3)	1	6	3	21	
Observational	5 (3.8)	0	2	0	3	
Sponsor						
Academic	69 (51.9)	6	15	6	42	0.276
Industrial	64 (48.1)	21	5	37	1	
Population						
Only children	56 (42.1)	14	6	24	12	1.000
Children and adults	77 (57.9)	13	14	19	31	
Sex						
Female	1 (0.8)	0	0	0	1	0.158
Male	20 (15.0)	2	2	13	3	
Both	112 (84.2)	25	18	30	39	
Methodology						
Randomized	50 (37.6)	20	7	20	3	0.000*
Non-randomized	83 (62.4)	7	13	23	40	
Multicentric						
Yes	86 (64.6)	24	7	39	16	0.852
No	47 (35.4)	3	13	4	27	
Sample size calculation						
Yes	66 (49.6)	17	8	21	20	0.589
No	67 (50.4)	10	12	22	23	
Mention of necessary items for sample size calculation						
Yes	57 (42.9)	15	9	19	14	0.200
No	76 (57.1)	12	11	24	29	
Interim analysis						
Yes	21 (15.8)	5	2	12	2	1.000
No	112 (84.2)	22	18	31	41	
Subgroup analysis						
Yes	26 (19.5)	9	2	10	5	0.494
No	107 (80.5)	18	18	33	38	
Intention to treat analysis						
Yes	54 (40.6)	19	7	19	9	0.016*
No	79 (59.4)	8	13	24	34	

The *p* value corresponds to the comparison of the two periods for each listed variable including both “drug” and “non drug” RCTs

Data are n (%) of protocols; *p-value <0.05

**Table 2:** Characteristics of randomized controlled trials

Characteristics of RCTs	*n* = 50	2003–2008 (6 y) *n* = 27		2009–2014 (6 y) *n* = 23		
	Total n (%)	Drug 20 (74)	Non drug 7 (26)	Drug 20 (87)	Non drug 3 (13)	
Experimental design						
Parallel	43 (86)	19	5	17	2	0.697
Cross-over	7 (14)	1	2	3	1	
Level of blind						
Double blind	29 (58)	13	1	14	1	0.052
Single blind	2 (4)	1	0	0	1	
Open labeled	19 (38)	6	6	6	1	
Allocation concealment						
Inadequate	4 (8)	2	0	2	0	0.055
Unclear	20 (40)	7	6	5	2	
Adequate	26 (52)	11	1	13	1	
Jadad class						
* Median [interquartile range]*		*4 [3–5]*	*1 [1–2]*	*5 [2.5–5]*	*3 [1–5]*	0.758
Low quality <3	15 (30)	3	6	5	1	
High quality ≥3	35 (70)	17	1	15	2	
Jadad class in sensitivity analysis						
* Median [interquartile range]*		*5 [3–5]*	*3 [1–5]*	*5 [4–5]*	*5 [5–5]*	0.650
Low quality <3	5 (15)	2	1	2	0	
High quality ≥3	28 (25)	13	1	12	2	

Data are n (%) of RCTs and median [1st quartile – 3rd quartile] of Jadad score

The *p* value corresponds to the comparison of the two periods for each listed variable including both “drug” and “non drug” RCTs

The time periods before and after the regulation and the respective number of protocols were the following: 2003–2008 (*n* = 47) and 2009–2014 (*n* = 86). The number of submitted pediatric protocols doubled between the two periods. We observed a statistically non-significant trend toward an increase in the number of academic protocols over time. RCTs accounted for 38% (50/133) of the studies submitted between 2003 and 2014. There was no significant change between the two periods except for the percentage of randomized studies that seem less substantial after the new regulation on pediatric medicines and for the intention to treat analysis (Table 1).

**Fig. 1:**
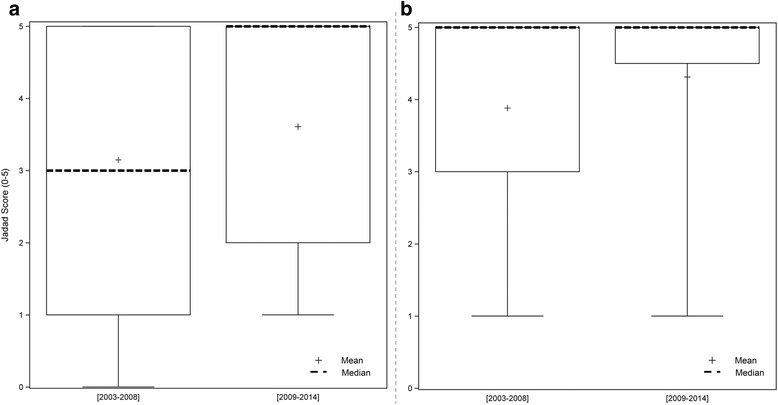
Boxplots representing the Jadad score by time periods: **a** All RCTs; **b** sensitivity analysis

The overall median Jadad score of RCTs was high (period 2003–2008: 3 [1–5]; period 2009–2014: 5 [2–5]). Overall, 70% (35/50) of the protocols had a “high” quality score, and 30% (15/50) a “low” quality score (Table 2). The analysis of the evolution of Jadad scores over time showed a quality trend that was statistically non-significant (Fig. 1).

### Sensitivity analysis

Among the 50 RCT protocols, 29 were double blind and 21 were not. Among these 21 open-label protocols, the blinding of the investigator and the patient was not feasible in 17 RCTs. These open design conditions were considered as “justified” and were removed from the sensitivity analysis. We noted that of the 17 justified open-label RCTs, only two had an independent outcome assessor. Among the 33 remaining RCTs, 15% (5/33) were “low” quality vs 85% (28/33) “high” quality RCTs (NS). The median Jadad score, increased from 3.5 to 5 but the quality trend with time remained statistically non-significant.

### Results from data banks

**Fig. 2:**
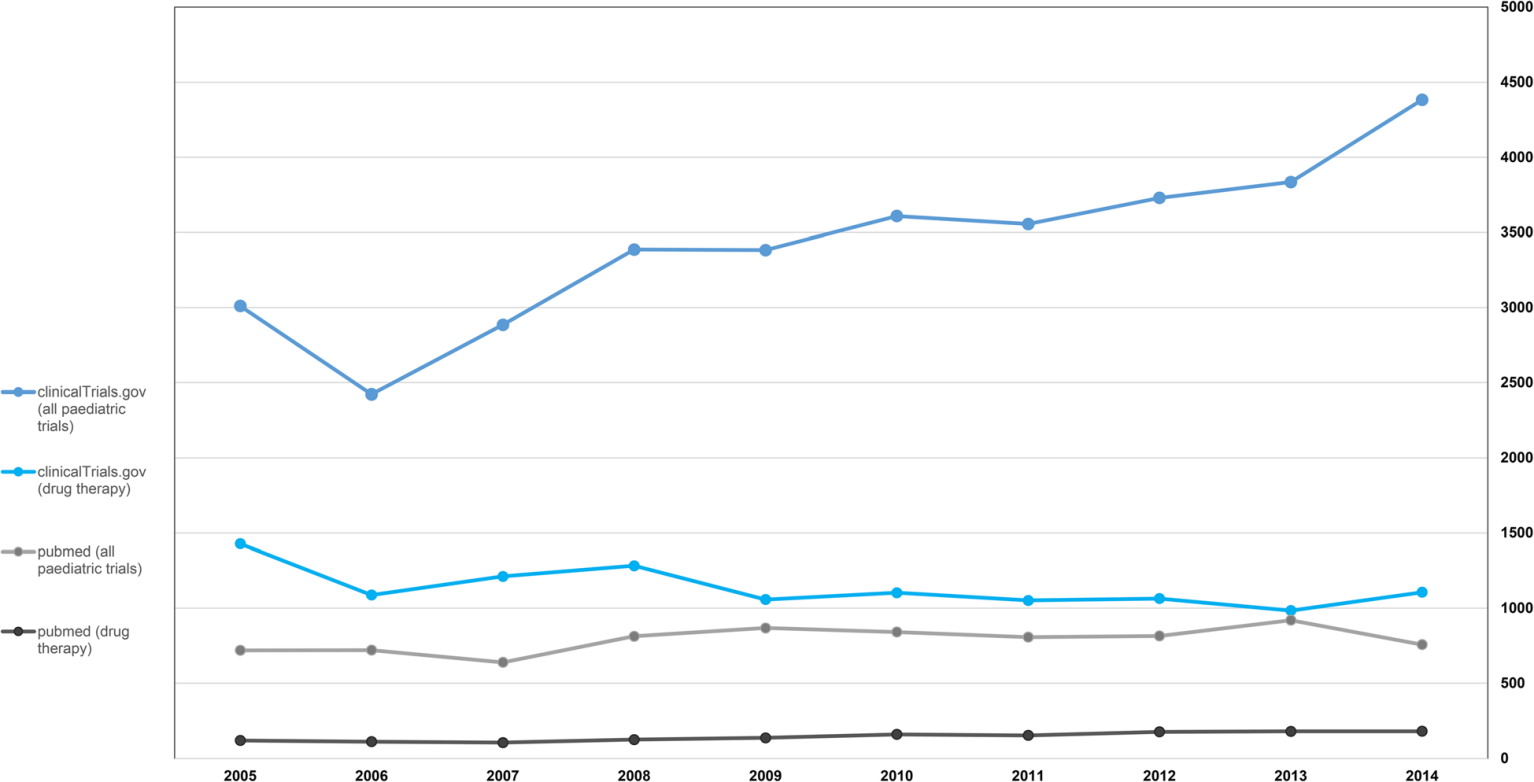
Results from data banks

The results from the Medline and ClinicalTrials.gov search are presented in Fig. 2.

## Discussion

We assessed how the quantity ands quality of pediatric clinical trial protocols submitted to a French IRB, has changed over a decade that has seen the introduction of the EU Pediatric Regulation. We observed that the number of submitted pediatric protocols doubled the last six years. We also observed a trend toward an increase in the number of institutionally sponsored protocols. Yet, it is not clear whether the increase in the number of institutionally protocols has been influenced by the European regulation, as institutions did not receive any incentive for performing RCTs, and are not seeking marketing authorization as a result of their clinical trials. Altogether it seems that the introduction of the new regulation had no impact on the quality of the trials, at least in our region. No significant improvement of the Jadad score for pediatric RCTs protocols submitted to our committee was detected.

In order to assess the impact of open label studies on the Jadad score, we performed a sensitivity analysis by excluding protocols with justified open-label approach. Our results showed that the mean Jadad score increases significantly and seems to be influenced by the open-label studies. However, the quality trend remains unchanged after the sensitivity analysis. Hrobjartsson et al. [31] assessed the impact of unblind outcome assessment on estimated treatment effects in RCTs with binary outcomes. They found that on average, non-blinded assessors of subjective binary outcomes generated substantially biased effect estimates in RCTs, exaggerating odds ratios by 36%. In our study, only 2 open-label trials had a blind assessment of the primary outcome. Thirteen had objective outcomes, such as death and bleeding, for which the blinding of the assessor might have a limited influence on the estimation of the treatment effect, and 3 protocols had a subjective outcome.

At the European level in its 2015 annual report (published on May 3rd, 2016), the European Commission reported that there was 340 pediatric trials authorized in 2006 on a total of 4272 (adults and/or children) trials (8%), and 763 pediatric trials authorized in 2015 on a total of 4242 trials (18%). This data was extracted from the protocol-related information in EudraCT [32].

Besides, in its 5-year interim report on the implementation of the European pediatric medicine regulation, the European Commission noticed an increase in the number of medicines available for children and an increase in the information related to the medicines used in children [33]. Since the implementation of the pediatric regulation, 31 out of 152 new medicines have been authorized for pediatric use.

We observed in ClinicalTrials.gov an increase in the number of pediatric studies currently registered over time. This result provide an accurate picture of the current pediatric trials. These findings are consistent with the increase of pediatric studies submitted to the Ethics Committee over the last decade.

There is evidence that the randomized trial literature reports favorable results leading to spins. A spin is defined as specific reporting that could distort the interpretation of the results and mislead the readers [34, 35]. Trial registration improved the transparency of clinical research but summary protocols do not encompass detailed information on the methods. Public access to trial protocols should not be limited to regulatory agency submissions that have led to positive decisions for market authorization, as many institutional non pharmacological trials are only assessed by IRBs. Confidentiality of protocol content is a common obstacle to the access to the protocols submitted to IRBs [36]. After authors signed a confidentiality agreement, our IRB gave them access to the protocols on site. After controlling the anonymization (withdrawal of the name of the drug and the sponsor) the IRB then authorized authors to hold an electronic database of the information gathered from the protocols. In the future, the access to IRB protocols could be facilitated by the global sharing of electronic databases for managing IRB protocols, as it is now the case in France, since 2016 [37, 38].

The number of reviews based on protocols remain, however, limited and difficult [39], suggesting that there is still a need for formal framework to facilitate access to protocols submitted to IRBs as underlined ten years ago [36]. It took more than twenty years before registration of protocol summary became the rule [40], under the impetus of the International Committee of Medical Journal Editors (ICMJE), and via a World Health Organization platform (International Clinical Trials Registry Platform) [40–42]. Unfortunately, public access to full protocols submitted to IRB is seemingly following the same long path. The new EU regulation No 536/2014 on clinical trials, entered into force in the second semester of 2016 after it had been harmonized with the new French law on research involving humans (Jardé law), aims to create an environment that is favorable for conducting clinical trials, with the highest standards of patient safety, for all EU Member States. The Jardé law has modified the method of allocating files to the Committee for the protection of persons, which is now done by central electronic randomization [37].

### Study limitations

Our study has some limitations. First, to assess the quality of protocols, we used the Jadad score, which does not take into account important methodological aspects such as the “intention to treat” analysis or the sample size calculation. Although other quality assessment tools for RCTs are available, the Jadad scale is considered as the most reproducible and is used in many meta-epidemiological studies [43]. Secondly, our results are restricted to one IRB that might not be representative of other parts of France or Europe. More data coming from others countries are therefore mandatory to confirm our findings. In addition, our sample size was small and our analyses were underpowered.

Although our results are not representative of the overall European pediatric clinical research, they indicate that in our IRB, the European regulation did not influence the number of pediatric studies sponsored by pharmaceutical industries nor did influence the quality of the submitted protocols. We believe that, in order to improve the quality of protocols regulators and sponsors should refer to the SPIRIT checklist specific to the writing of protocols and that we should promote the access to protocol [30].

### Future directions

There is still room for improvement of the quality of clinical research for children. IRBs have an important role in promoting high quality studies for children and to avoid wasting by preventing unnecessary and unsound studies.

## Conclusion

Independently of the lack of impact on the quality, a progressive increase on the overall number of pediatric protocols submitted to the ethic committee is observed. However, it is difficult to find a clear link between this increase and the European Pediatric Regulation implementation.

## Abbreviations

EQUATOR: Enhancing the Quality and Transparency Of health Research
ICMJE: International Committee of Medical Journal Editors
IRB: Institutional Review Board
RCT: Randomized Clinical Trial
SPIRIT: Standard Protocol Item: Recommendations for Interventional Trials
